# Association of Novel Mutations in the Vasoactive Intestinal Peptide Receptor-1 Gene with Egg Shell Thickness in Three Strains of Laying-Type Quail

**DOI:** 10.3390/ani15101373

**Published:** 2025-05-09

**Authors:** Xinle Wang, Huricha Chen, Ying Lei, Qiankun Wang, Gan Li, Junyan Bai

**Affiliations:** 1 College of Animal Science, Henan University of Science and Technology, Luoyang 471023, China; wangxinle163@163.com (X.W.);; 2 School of Tropical Agriculture and Forestry, Hainan University, Haikou 570228, China

**Keywords:** *VIPR-1*, polymorphism, egg quality, laying performance, quail

## Abstract

This study aimed to investigate the potential role of the vasoactive intestinal peptide receptor-1 (*VIPR-1*) gene polymorphisms and haplotypes in influencing egg production performance and egg quality parameters in laying-type quail. Genomic DNA was extracted from 150 quail across three strains: Chinese yellow (CY, *n* = 50), Beijing white (BW, *n* = 50), and Korean (KO, *n* = 50). We designed two pairs of primers and initiated PCR amplification, after which the amplified products were sent to a testing company for purification. Sanger sequencing was employed to identify single nucleotide polymorphisms (SNPs) within the *VIPR-1* gene. Two SNP sites were selected for genotyping; g.1603402T>G was analyzed using PCR-RFLP with the *Bsr*D I enzyme, while g.1614884A>G was genotyped using the *Hpy*CH4 IV enzyme. The association results revealed that the g.1603402T>G site showed significant association with egg shell thickness (EST) in the BW strain (*p* < 0.05). There were no significant associations between these two loci and the remaining egg quality traits in the BW and KO strains (*p* > 0.05). Differences in egg quality and laying performance among haplotype combinations were not significant (*p* > 0.05). In conclusion, the *VIPR-1* gene, with its identified polymorphisms and haplotypes, has potential as a molecular marker that could improve egg shell thickness in BW quail.

## Simple Summary

Quail are widely raised in China due to their low feeding costs, short production cycles, and high nutritional value. Based on their use, quail can be categorized into meat and egg-laying types. Egg-laying quail not only possess significant economic value but also hold important scientific research value. This study aimed to investigate the polymorphisms of the vasoactive intestinal peptide receptor-1 (*VIPR-1*) gene related to egg quality and laying performance in three distinct quail strains, using scientific breeding techniques to enhance breeding efficiency and promote sustainable development of the quail industry.

## 1. Introduction

In the wake of the COVID-19 pandemic and recurring health threats like influenza and mycoplasma pneumonia, there has been a heightened emphasis on adopting healthier diets to bolster immunity and reduce disease vulnerability [1]. Quail eggs are rich in nutrients, including essential minerals such as calcium, phosphorus and iron, as well as beneficial fatty acids such as phospholipids, making them a good choice for health-conscious consumers [2]. Additionally, quail, which are commonly reared in small-scale economic poultry farming in China have garnered increasing attention in recent years due to their advantages, including a shortened growth cycle, high reproductive capabilities, and substantial economic benefits compared to other poultry species [3]. Central to the profitability of quail farming are egg quality and laying performance, prompting scientific efforts to identify genetic markers associated with these productivity indicators.

As biotechnology continues to advance, molecular approaches leveraging various genetic markers and sequencing methods, such as mitochondrial DNA markers [4], microsatellite markers [5], restriction site-associated DNA sequencing [6] and whole-genome resequencing [7], have been widely used in the identification of SNPs and screening of candidate genes in animals. Despite the emergence of new technologies, SNPs, recognized as the third generation of molecular markers, remain extensively utilized in animal breeding due to their high genetic stability, abundance of polymorphic sites, ease of detection, and low cost. The vasoactive intestinal peptide receptor-1 (*VIPR-1*) gene, a glycoprotein integral to the glucagon/VIP receptor family and linked to Gs proteins, plays a pivotal role in regulating quail physiology and productivity. In birds, *VIPR-1* mediates the action of the vasoactive intestinal peptide (*VIP*), a neuropeptide critical for hypothalamic–pituitary–gonadal (HPG) axis regulation [8]. *VIP* binding to *VIPR-1* stimulates adenylate cyclase activity, triggering cAMP-dependent signaling pathways that influence gonadotropin synthesis (e.g., follicle-stimulating hormone and luteinizing hormone) and ovarian follicular development—key drivers of ovulation and egg-laying frequency [9]. This receptor’s structure features a large hydrophilic domain at the extracellular N-terminus, seven highly conserved hydrophobic transmembrane helices, and a cytoplasmic C-terminus [10], enabling its versatile interaction with ligands and downstream effectors. Studies have validated *VIPR-1*’s importance in economically pertinent traits; for instance, Zhou et al. [11] linked the C+598T and C+53327T variants to chicken brooding behavior, suggesting a potential role in maternal incubation-related hormonal modulation. Pu et al. [12] expanded upon this, demonstrating associations between G373T and A313G mutations, *VIPR-1* diplotypes, and variations in egg production and size in laying quail. These findings align with *VIPR-1*’s proposed function in modulating ovarian steroidogenesis and follicular maturation, processes directly tied to egg output and quality. This underscores *VIPR-1*’s conserved role across avian species in regulating both reproductive output and growth efficiency.

Given the paucity of research on *VIPR-1* polymorphism impacts on quail egg quality and laying performance, our study employed Sanger sequencing and PCR-RFLP methodologies to thoroughly examine *VIPR-1* gene variations. Beijing white quail (BW), Chinese yellow quail (CY), and Korean quail (KO), as common egg-type quail breeds, have an important position in China’s quail breeding industry [13]. BW quail have the advantage of higher egg production; CY quail are more resistant to cold and disease [14]. KO quail exhibit a relatively fast early growth rate and have higher uniformity in developmental traits such as body weight and size compared to other quail populations. These characteristics provide an important genetic basis for the improvement and cultivation of quail breeds in China [15]. Each of them has unique advantages and meets the requirements of different market demands and breeding environments. We investigated the association of these polymorphisms with egg quality and laying performance in the three quail breeds. We aimed to establish a robust dataset highlighting the associations of *VIPR-1* gene polymorphisms with crucial production traits in quail, thereby guiding advancements in quail breeding science.

## 2. Materials and Methods

### 2.1. Ethical Treatment

All animal experiments strictly adhered to the Guidelines for Experimental Animals issued by the Ministry of Science and Technology (Beijing, China). Research on live animals was approved by the local Institutional Animal Care and Use Committee through the use of appropriate management and laboratory techniques to avoid unnecessary discomfort to animals (No. 2021034). Written informed consent was obtained from the owners for the participation of animals in this study.

### 2.2. Experimental Populations and Phenotypic Data Collection

In this study, 150 egg-type quail (50 healthy females each from the CY, BW, and KO strains) were procured from Henan University of Science and Technology Quail Breeding Co., Ltd., situated in Luoyang, Henan, China. The quail were housed in a clean, well-ventilated environment under uniform conditions at our institution’s experimental farm. Throughout the rearing period, the quail were granted ad libitum access to food and water. Supplementary heating was administered during the first two weeks to maintain optimal thermal conditions. The ambient temperature and humidity within the quail house was meticulously controlled, with specific details provided by Bai et al. [16]. At 40 days of age, each quail was transferred to individual cages for rearing. Quail diets were phased from high-energy (2900 kcal/kg ME), high-protein (24%) formulations during growth to reduced-energy (2800 kcal/kg ME), lower-protein (20%) regimens for laying periods.

As quail transition from growth to sexual maturity and egg production, they undergo three primary egg-laying phases: the early stage (approximately 9 weeks of age), middle stage (14 weeks of age), and peak stage (18 weeks of age). During the peak laying period (≈18 weeks), three eggs were collected from each of the 50 quail per strain (BW, CY, KO), resulting in 150 eggs per strain (3 eggs × 50 quail) and 450 eggs total for analysis. Egg quality and laying performance measurements were first averaged within individual quail (3 eggs per quail → 1 mean value per quail). Egg production and quality measurements included egg weight (EW), egg longitudinal diameter (ELD), egg horizontal diameter (EHD), egg shape index (ESI), egg yolk weight (EYW), egg yolk height (EYH), egg yolk diameter (EYD), egg yolk index (EYI), eggshell thickness (EST), and albumen height (ALH). Additionally, laying performance metrics for the three strains at 18 weeks of age were evaluated, including feed-to-egg ratio (F/E), egg production (EP), total egg mass (TEM), average egg size (AES), and laying rate (LR).

### 2.3. DNA Sample Collection, Primers, and Polymerase Chain Reaction

We collected 2 mL of blood from the subwing vein of each quail in anticoagulant tubes and stored it at −80 °C for subsequent extraction of genomic DNA. DNA was extracted using the Poultry Whole Blood Genome Extraction Kit (BioTeKe, Wuxi, China), following the manufacturer’s protocol. The purity and concentration of genomic DNA determined by NanoDrop 2000 of Thermo Fisher Scientific (Waltham, MA, USA) showed an absorbance at OD260 nm/OD280 nm of between 1.8 and 2.0 [17]. For amplification of the quail *VIPR-1* gene, primer pairs targeting exon 4–5 (F: 5′-GCGTTCTATGGCACAGTTA-3′; R: 5′-AAAGCAATGTTCGGGTTCT-3′) and exon 6–7 (F: 5′-GCTGCTGGTGGAAGGGTTA-3′; R: 5′-CCGTCCAAGCAGTGATGAA-3′) were designed as described previously [12]. The fragments were amplified in a 25 µL reaction volume consisting of 12.5 µL of the 2× Taq Mix (0.1 U/µL), 1 µL of genomic DNA (50 ng/µL), 9.5 µL of ddH_2_O, and 1 µL of each forward and reverse primer (4 μM). The PCR amplification was conducted according to the following procedure: initial denaturation at 94 °C for 4 min, 32 cycles of denaturation at 94 °C for 40 s, annealing at 60.3 °C and 61.9 °C for 1 min, and extension at 72 °C for 1 min 20 s, followed by a final extension at 72 °C for 10 min. The annealing temperature in the thermal cycler is determined by setting up gradient experimental screening.

### 2.4. Sequencing and PCR-RFLP Analysis

We randomly selected 10 Chinese yellow quail, 10 Beijing white quail, and 10 Korean quail (from a larger dataset of quail with complete egg production and quality measurements) for two-direction pooled DNA sequencing. The PCR product was sent to Tsingke Biotech Ltd. (Beijing, China) for purification and then sequenced. Sequence alignment was performed using the Blast program (1.4.0) in the NCBI database and BioEdit 7.2 software (Informer Technologies, Inc., Roseau Valley, Dominica) [18] to determine the location of the SNP sites (Genbank accession number: LSZS01000597.1). Subsequently, *Bsr*D I and *Hpy*CH4 IV enzymes were screened from the WatCut (“http://heimanlab.com/cut2.html (accessed on 18 April 2021)” based on the type and location of the SNP. A total of 143 samples were subjected to genotyping using the PCR-RFLP method. The digestion of PCR products was performed using reaction conditions as described in a previous study [16].

### 2.5. Statistical Analysis

The genotypes were determined based on imaging results, and genotypes and alleles were recorded in 143 quail. The Arlequin (version 3.52) was utilized to perform the Hardy–Weinberg equilibrium test (HWE) and to calculate expected heterozygosity (He), observed heterozygosity (Ho), effective allele numbers (Ne), and polymorphism information content (PIC) [19]. Finally, we employed the generalized linear model procedure in SPSS (version 26.0; IBM Corp., Armonk, NY, USA) to analyze the association between SNPs (or haplotypes) within the VIPR-1 gene regions of exon 4 to 5 and exon 6 to 7 and egg quality and laying performance. First, the normality of the data distribution within each group was assessed using the Shapiro–Wilk test (*p* < 0.05). Subsequently, the homogeneity of variances across groups was verified via Levene’s test (*p* < 0.05). For normally distributed data with equal variances, one-way ANOVA was employed to evaluate overall group differences. If the ANOVA F-test reached significance (*p* < 0.05), Tukey’s HSD post hoc test was applied for pairwise comparisons, controlling the family-wise error rate. In cases of violated variance homogeneity (Levene’s *p* < 0.05) and non-normally distributed data (Shapiro–Wilk *p* < 0.05), the non-parametric Kruskal–Wallis H test was conducted, followed by pairwise comparisons with Dunn–Bonferroni adjustment. When the ANOVA performed for each group of genotypes showed a significant difference (*p* < 0.05), the statistical difference between the two genotypes was subsequently evaluated using Bonferroni. The results were expressed as means ± standard error (SE). The specific model used for the association analysis was as follows:

$$Y_{ij}=\mu +G_{i}+e_{ij}$$

where $Y_{ijk}$ is the phenotypic value of egg quality or laying performance, μ is the overall mean value, $G_{i}$ is the effect of the genotype or haplotype, and $e_{ij}$ is the random error.

## 3. Results

### 3.1. Descriptive Statistics

**Figure 1:**
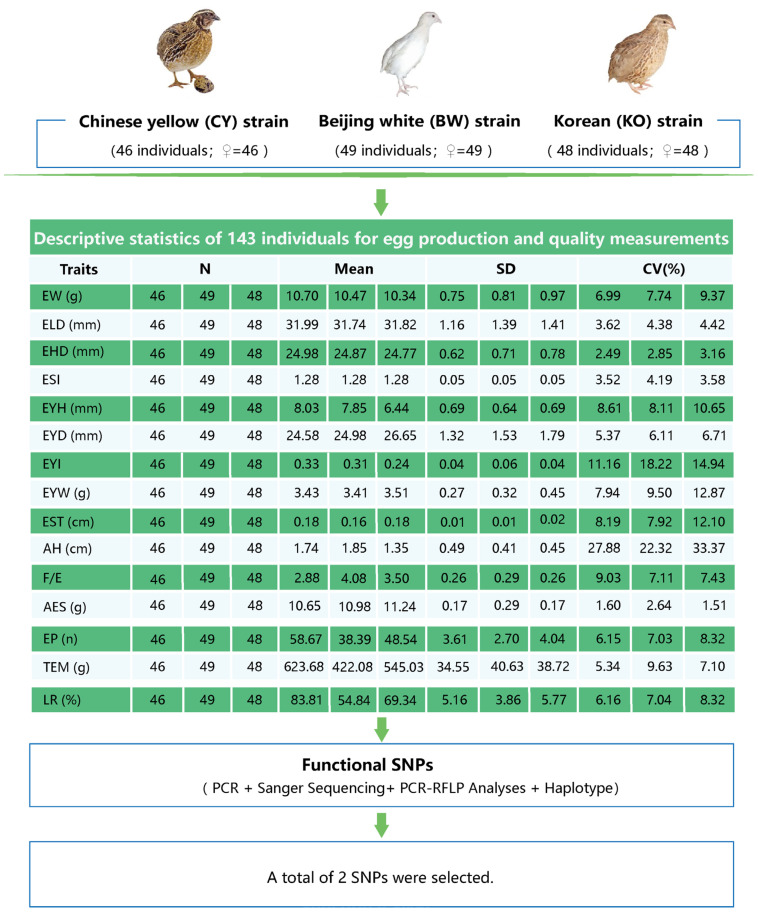
Quail egg production and quality measurements used in this study. Abbreviations: EW, egg weight; ELD, egg longitudinal diameter; EHD, egg horizontal diameter; ESI, egg shape index; EYH, egg yolk height; EYD, egg yolk diameter; EYI, egg yolk index; EYW, egg yolk weight; EST, egg shell thickness; AH, albumen height; F/E, feed to egg ratio; EP, egg production; TEM, total egg mass; AES, average egg size; LR, laying rate.

Figure 1 shows the details of the egg production and quality measurements of the three strains of laying-type quail. A total of 143 female quail were recorded for egg quality and laying performance traits. The coefficient of variation for AH was the highest for KO at 33.37%, and the coefficient of variation for EHD was the lowest for CY at 2.49%. This figure underscores the extent of variability within the quail strains, which is pivotal for genetic evaluation and improvement programs.

### 3.2. Polymorphism of the VIPR-1 Gene in Quail

**Figure 2:**
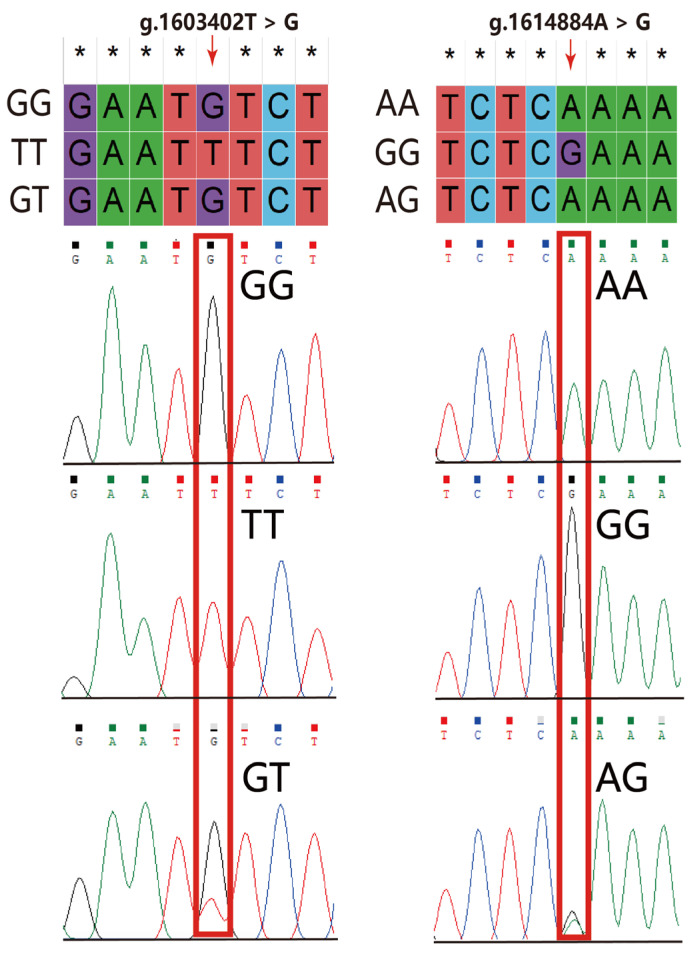
Sequencing results of g.1603402T>G and g.1614884A>G sites. *: sites that are not mutated; red box: sites that are mutated.

**Figure 3:**
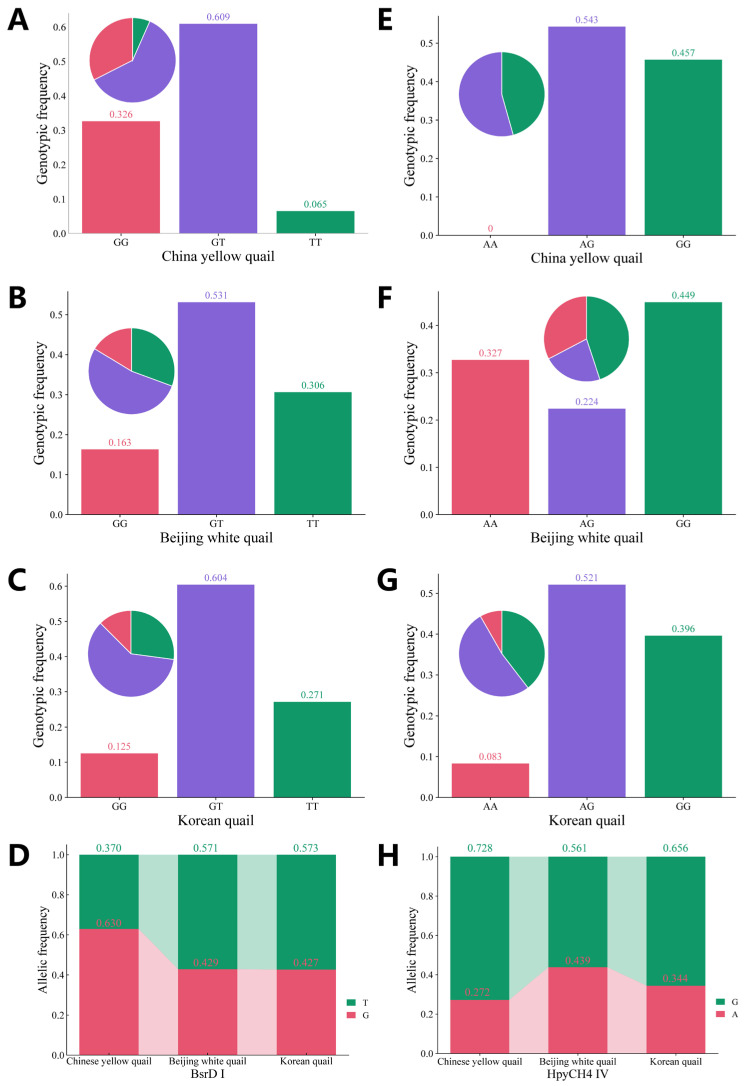
Genotype frequency and allele frequency of g.1603402T>G and g.1614884A>G sites. (**A**) Genotypic frequency of g.1603402T>G site in China yellow quail population; (**B**) Genotypic frequency of g.1603402T>G site in Beijing white quail population; (**C**) Genotypic frequency of g.1603402T>G site in Korean quail population; (**D**) Allelic frequency of g.1603402T>G site in three quail populations; (**E**) Genotypic frequency of g.1614884A>G site in China yellow quail population; (**F**) Genotypic frequency of g.1614884A>G site in Beijing white quail population; (**G**) Genotypic frequency of g.1614884A>G site in Korean quail population; (**H**) Allelic frequency of g.1614884A>G site in three quail populations.

A total of 20 SNP sites were identified in the *VIPR-1* segment exon 4 to 5 region by Sanger sequencing. Only the g.1603402T>G site can be digested by the *Bsr*D I enzyme (GCAATG↓NN) as an enzyme restriction site. Additionally, 15 SNP sites were found in the *VIPR-1* segment exon 6 to 7 region by Sanger sequencing. Only the g.1614884A>G can be digested by the *Hpy*CH4 IV enzyme (TG↓CA) for detecting different genotypes. Sequencing results of g.1603402T>G and g.1614884A>G sites are shown in Figure 2. The PCR digestion products are shown in Figure S1, and three genotypes were detected at both the g.1603402T>G and g.1614884A>G sites. The population genetic information of the two sites is presented in Table 1, and genotype frequency and allele frequency are presented in Figure 3. The GT genotype frequency at the g.1603402T>G site was the highest in CY, BW, and KO, with frequencies of 60.9, 53.1, and 60.4%, respectively. Moreover, allele G (63.0%) had the highest frequency at the g.1603402T>G site in CY, and allele T (57.1 and 57.3%) had the highest frequency at the g.1603402T>G site in BW and KO. The AG genotype frequency at the g.1614884A>G site was highest in CY and KO, with frequencies of 54.3 and 52.1%, respectively. The GG genotype frequency at the g.1614884A>G site was highest in BW, with a frequency of 44.9%. Furthermore, the G allele (72.8, 56.1, and 65.6%) was the most frequent allele at the g.1614884A>G site in CY, BW, and KO, respectively (Figure 3). The two sites were in moderate polymorphism (0.25 < PIC < 0.50) in CY, BW, and KO. The g.1603402T>G site was under HWE in BW and KO (*p* > 0.05). Conversely, the g.1614884A>G site significantly deviated from HWE (*p* < 0.05) in CY and BW. The g.1614884A>G site was in HWE (*p* > 0.05) in KO, enabling reliable subsequent association analysis.

### 3.3. Association Analysis of SNPs and Haplotype Combinations with Egg Quality in Quail

**Figure 4:**
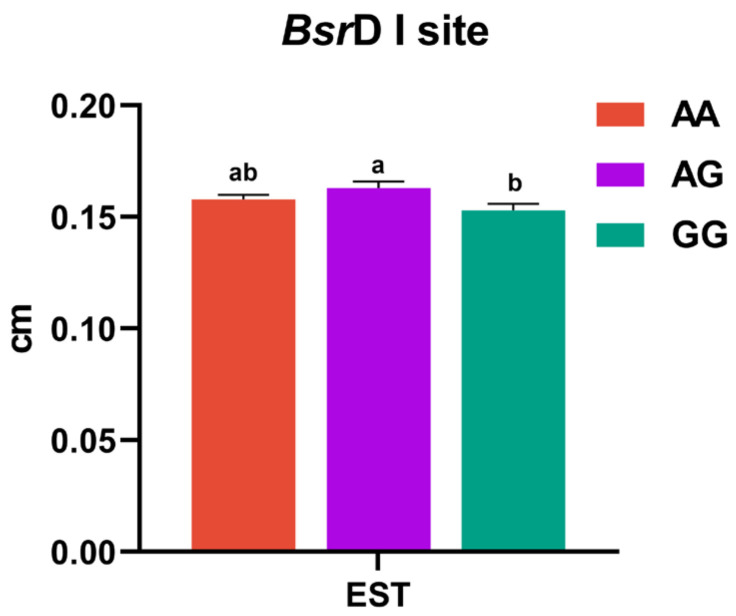
Association analysis of the *Bsr*D I site of *VIPR-1* gene with egg quality trait in BW quail. Note: EST, egg shell thickness. ^ab^ The difference between genotypes with different lowercase letters was significant (*p* < 0.05).

The g.1603402T>G site was not in HWE in the CY population, and the g.1614884A>G site was not in Hardy–Weinberg Equilibrium (*p* < 0.05) in both the CY and BW populations. Therefore, they were not analyzed for associations. In order to leave high-quality SNPs, only the data on laying performance and egg quality of g.1603402T>G in BW and KO quail as well as g.1614884A>G in the KO population were analyzed for associations. The g.1603402T>G site had a significant association with EST in the BW population. The TG genotype of the g.1603402T>G site showed significantly higher EST in BW (*p* < 0.05, Figure 4). There were no significant differences among genotypes for the remaining nine egg quality traits in the BW population (*p* > 0.05, Table S1). Differences among genotypes for the 10 egg quality traits in the KO population were not significant (*p* > 0.05, Table 2). Specific information is in Table S2. The g.1614884A>G locus was not significantly associated with any quail egg quality traits in the KO population (*p* > 0.05, Table 3). Haplotypes were constructed for the two loci present in the KO population. The results of the association analysis between the haplotype combinations and the quail egg quality traits showed that the differences between the haplotype combinations for each trait were not significant (*p* > 0.05, Table 4).

### 3.4. Association Analysis of SNPs and Haplotype Combinations with Laying Performance in Quail

The association analysis of *VIPR-1* gene SNP with quail egg laying performance is shown in Table 5, Table 6 and Table 7. The results of the normal distribution test (Shapiro–Wilk test) showed that the genotypes of each trait at the g.1603402T>G locus of BW and KO strains did not conform to a normal distribution (*p* < 0.05, Tables S5–S8). Therefore, a non-parametric test (Kruskal–Wallis test) was used, which showed non-significant differences between the genotypes of each trait (*p* > 0.05, Table 5). The results of the KO strain for the g.1614884A>G locus showed the same result. The genotypes did not indicate significant differences from each other (*p* > 0.05, Table 6) nor between the haplotype combinations (*p* > 0.05, Table 7).

## 4. Discussion

The egg quality and laying performance in egg-type quail directly influence breeding efficiency and market value [20]. Laying performance, a core economic trait, is as critical as egg quality, encompassing total egg production, cycle stability, peak period duration, and feed conversion efficiency [21]. In view of this, integrating egg quality and laying performance optimization into genetic improvement and breeding management has become central to advancing the quail egg industry toward sustainability and high productivity.

The two SNP loci identified in this study were highly polymorphic in three quail lines and showed different gene frequencies in different populations. Pu et al. [12] detected two variation sites (G373T and A313G) of the *VIPR-1* gene in three egg-laying quail lines, suggesting that the gene is relatively rich in polymorphisms. In the context of the *VIPR-1* gene, these SNPs may contribute to differences in egg-laying performance among quail strains. Since these two SNPs conformed to HWE only in the BW and KO populations, we performed an association analysis of the two loci with these two populations. For the association analysis, we applied Dunn–Bonferroni adjustment for correction and performed strict controls to prevent false positive results. Association analysis includes not only the effects of individual SNP loci, but also how the constructed haplotype combinations further explain the variation in egg quality. These findings not only enhance our understanding of quail genetics, but also provide a scientific basis for future molecular marker-assisted selection to improve productivity and product quality in the egg industry [22]. The g.1603402T>G polymorphism had a significant association with EST in the BW population, with TG genotypes demonstrating significantly higher values (*p* < 0.05). Different strains of quail have accumulated different genetic variations during long-term selection and evolution. Each line has its unique genetic background, including specific gene combinations and allele frequency distributions. These genetic differences may result in different levels of association between SNPs and traits of specific strains of quail. Consistent with our findings, a previous study on laying quail by Pu et al. [12] claimed a significant association between two SNPs located in the *VIPR-1* gene (G373T and A313G) and egg-laying traits. Zhou et al. [11] showed that the C+598T site located in intron 2 of the *VIPR-1* gene might be associated with broody frequency (%), while another C+53327T site was significantly associated (*p* < 0.05) with duration of broodiness. A study by Bai et al. [16] using Savimalt and French Giant meat quail showed that the *Bsr*D I and *Hpy*CH4 IV loci were significantly associated with growth traits in quail, inferring that the *VIPR-1* gene could be used as a molecular marker to improve their growth traits. Nguyen et al. [23] identified variants of C1715301T at 486 bp by *VIPR-1*/*Taq* I and C1704887T at 434 bp by *VIPR-1*/*Hha* I in Noi chickens in Vietnam. They found significant association between genotypes and egg numbers (*p* < 0.05) in 20 weeks of laying (28–47 weeks of age) in Noi chicken. This underscores *VIPR-1*’s conserved role across avian species in regulating both reproductive output and growth efficiency. Collectively, these findings highlight the *VIPR-1*’s potential as a key regulator of poultry economics, informing genetic improvement strategies. Taken together, these studies suggest that the *VIPR-1* gene plays a crucial role in influencing egg-laying traits in quail. The identification of specific SNPs and their associations with phenotypic traits provides valuable genetic markers for improving egg production in these birds through targeted breeding programs.

Constructing haplotypes from candidate regions identified by significant loci can better aid in finding genes or loci associated with a trait, with greater efficacy compared to individual SNP markers. These analyses serve as a robust validation of the identified SNPs, adding credibility to genetic research findings [24]. In the KO strain, haplotypes did not significantly influence the traits under consideration (*p* > 0.05). The *VIPR-1* gene has been extensively studied in the context of egg-laying traits in Japanese quail. In a prior study on Japanese quail, it was found that seven diplotypes derived from four haplotypes (GG, GT, TA, and GA) of the *VIPR-1* gene exhibited significant associations with age at first laying, egg weight, and egg number at 20 weeks of age [12]. In a study by Bai et al. [16], it was demonstrated that four diplotypes (A1A1, A1A2, A1A3, and A3A3) derived from the *VIPR-1* gene were significantly associated with BW, CW, CD, SL, BL and TC. Xu et al. [25] reported that C1704887T and C1715301T located in the *VIPR-1* gene were significantly associated with chicken egg number at 300 days of age. Haplotype analysis based on two mutations of the *VIPR-1* gene also validated this result. Chickens with haplotype combination (H1H3) had the largest number of eggs at 300 days of age (*p* < 0.05). In contrast to our study, two potential SNPs (C17687T and A17690T) located in the *VIPR-1* gene were reported to associate with egg number, total egg weight, and laying period in turkey hens [26]. The likely reason for this is that we applied a slightly more rigorous statistical methodology that filtered out false positives and ultimately produced more accurate results relative to the previous study. Our findings showed that the presence of polymorphic loci (g.1603402T>G site) in the *VIPR-1* gene were significantly associated with egg shell thickness in BW quail.

## 5. Conclusions

In summary, our results demonstrated a significant association between the Chr:2_g.1603402T>G of the *VIPR-1* gene and egg shell thickness in Beijing white quail. However, to fully understand the genetic architecture underlying quail performance and its economically significant traits, further research is warranted. This includes expanding the experimental population and incorporating additional genetic markers to conduct genome-wide association studies using whole-genome resequencing. Such studies will identify more molecular genetic markers significantly linked to economically important traits and validate the authenticity of these loci. Moreover, elucidating the molecular mechanisms underlying the effects of these SNPs and haplotypes on economically significant traits will enable exploration of their potential applications in genetic selection and breeding.

## Figures and Tables

**Table 1:** Genetic diversity parameters and Hardy–Weinberg Equilibrium (HWE) test results for g.1603402T>G and g.1614884A>G loci in CY, BW, and KO quail populations.

SNP	S	HWE		Ho	He	PIC	Ne
		***χ*2**	***p***				
g.1603402T>G	CY (46)	4.315	0.038	0.609	0.466	0.357	1.873
	BW (49)	0.340	0.560	0.531	0.490	0.370	1.960
	KO (48)	2.642	0.104	0.604	0.489	0.370	1.958
g.1614884A>G	CY (46)	6.405	0.011	0.543	0.396	0.317	1.655
	BW (49)	14.511	0.000	0.224	0.493	0.371	1.970
	KO (48)	1.144	0.285	0.521	0.451	0.349	1.822

Note: BW, Beijing white quail; CY, Chinese yellow quail; KO, Korean quail; He, expected heterozygosity; Ho, observed heterozygosity; HWE, Hardy–Weinberg Equilibrium test; Ne, effective allele numbers; PIC, polymorphism information content; S, Strain; SNP, single nucleotide polymorphism.

**Table 2:** Association analysis of *Bsr*D I site of *VIPR-1* gene with egg quality in quail.

S	EQ	*Bsr*D I (Mean ± SE)			
		GG	GT	TT	ANOVA/Kruskal–Wallis *p*-Value
BW (49)	EW (g)	10.50 ± 0.17	10.41 ± 0.18	10.55 ± 0.22	0.877
	ELD (mm)	31.8 ± 0.46	31.7 ± 0.29	31.6 ± 0.35	0.960
	EHD (mm)	24.8 ± 0.12	24.8 ± 0.16	24.9 ± 0.16	0.742
	ESI	1.28 ± 0.02	1.28 ± 0.01	1.26 ± 0.01	0.833
	EYH (mm)	7.80 ± 0.17	7.76 ± 0.11	8.01 ± 0.20	0.500
	EYD (mm)	25.33 ± 0.27	24.992 ± 0.29	25.78 ± 0.49	0.575
	EYI	0.26 ± 0.039	0.31 ± 0.006	0.32 ± 0.011	0.223
	EYW (g)	3.36 ± 0.06	3.40 ± 0.06	3.43 ± 0.10	0.888
	EST (cm)	0.15 ± 0.002 ^ab^	0.16 ± 0.003 ^a^	0.15 ± 0.003 ^b^	0.043
	AH (cm)	1.88 ± 0.10	1.82 ± 0.08	1.86 ± 0.11	0.922
KO (48)	EW (g)	10.40 ± 0.51	10.25 ± 0.18	10.47 ± 0.24	0.795
	ELD (mm)	31.8 ± 0.6	31.68 ± 0.2	32.09 ± 0.3	0.697
	EHD (mm)	24.8 ± 0.4	24.72 ± 0.1	24.82 ± 0.2	0.896
	ESI	1.28 ± 0.01	1.28 ± 0.01	1.293 ± 0.01	0.773
	EYH (mm)	6.63 ± 0.13	6.38 ± 0.15	6.47 ± 0.11	0.776
	EYD (mm)	25.95 ± 0.70	26.65 ± 0.36	26.96 ± 0.43	0.533
	EYI	0.25 ± 0.01	0.24 ± 0.01	0.24 ± 0.01	0.316
	EYW (g)	3.45 ± 0.20	3.47 ± 0.08	3.63 ± 0.13	0.568
	EST (cm)	0.17 ± 0.01	0.17 ± 0.01	0.17 ± 0.00	0.825
	AH (cm)	1.31 ± 0.18	1.32 ± 0.07	1.41 ± 0.15	0.939

Note: S, Strain; BW, Beijing white quail; KO, Korean quail; EQ, egg quality; EW, egg weight; ELD, egg longitudinal diameter; EHD, egg horizontal diameter; ESI, egg shape index; EYH, egg yolk height; EYD, egg yolk diameter; EYI, egg yolk index; EYW, egg yolk weight; EST, egg shell thickness; AH, albumen height. Homogeneity of variances was confirmed via Levene’s test (*p* > 0.05). One-way ANOVA followed by Tukey’s HSD post hoc test was applied. ^ab^ The differences between genotypes with different lowercase letters were significant (*p* < 0.05).

**Table 3:** Association analysis of *Hpy*CH4 IV site of *VIPR1* gene with egg quality in quail.

S	EQ	*Hpy*CH4 IV (Mean ± SE)			
		AA	AG	GG	ANOVA/Kruskal–Wallis *p*-Value
KO (48)	EW (g)	10.00 ± 0.33	10.44 ± 0.20	10.26 ± 0.23	0.665
	ELD (mm)	30.6 ± 0.5	32.1 ± 0.3	31.7 ± 0.2	0.154
	EHD (mm)	24.8 ± 0.3	24.7 ± 0.1	24.7 ± 0.2	0.975
	ESI	1.23 ± 0.02	1.29 ± 0.01	1.28 ± 0.01	0.056
	EYH (mm)	6.52 ± 0.35	6.56 ± 0.12	6.26 ± 0.17	0.367
	EYD (mm)	26.72 ± 1.07	26.71 ± 0.29	26.55 ± 0.50	0.956
	EYI	0.24 ± 0.02	0.24 ± 0.01	0.23 ± 0.01	0.735
	EYW (g)	3.67 ± 0.25	3.60 ± 0.07	3.36 ± 0.11	0.151
	EST (cm)	0.17 ± 0.01	0.17 ± 0.00	0.18 ± 0.01	0.155
	AH (cm)	1.49 ± 0.27	1.31 ± 0.08	1.36 ± 0.11	0.568

Note: S, Strain; KO, Korean quail; EQ, egg quality; EW, egg weight; ELD, egg longitudinal diameter; EHD, egg horizontal diameter; ESI, egg shape index; EYH, egg yolk height; EYD, egg yolk diameter; EYI, egg yolk index; EYW, egg yolk weight; EST, egg shell thickness; AH, albumen height.

**Table 4:** Association analysis of *VIPR-1* gene haplotype combinations with egg quality of Korean quail.

D	Traits (Mean ± SE)									
	EW (g)	ELD (mm)	EHD (mm)	ESI	EYH (mm)	EYD (mm)	EYI	EYW (g)	EST (cm)	AH (cm)
GGAG (4.2%)	11.40 ± 1.10	33.5 ± 1.2	25.5 ± 0.8	1.31 ± 0.01	6.70 ± 0.50	27.67 ± 0.02	0.24 ± 0.02	4.00 ± 0.20	0.17 ± 0.01	0.80 ± 0.10
GGGG (8.3%)	9.90 ± 0.46	31.1 ± 0.1	24.5 ± 0.5	1.26 ± 0.02	6.60 ± 0.07	25.09 ± 0.70	0.26 ± 0.01	3.17 ± 0.14	0.18 ± 0.01	1.56 ± 0.14
GTAA (4.2%)	10.20 ± 0.60	30.9 ± 0.1	25.0 ± 0.7	1.23 ± 0.03	6.30 ± 0.80	26.16 ± 2.29	0.24 ± 0.05	3.45 ± 0.45	0.17 ± 0.00	1.68 ± 0.05
GTAG (29.2%)	10.31 ± 0.27	31.7 ± 0.4	24.7 ± 0.1	1.28 ± 0.01	6.63 ± 0.19	26.64 ± 0.39	0.25 ± 0.01	3.62 ± 0.08	0.17 ± 0.01	1.35 ± 0.12
GTGG (27.1%)	10.20 ± 0.28	31.6 ± 0.3	24.6 ± 0.2	1.28 ± 0.01	6.12 ± 0.25	26.73 ± 0.64	0.23 ± 0.01	3.32 ± 0.14	0.18 ± 0.01	1.24 ± 0.10
TTAA (4.2%)	9.80 ± 0.50	30.3 ± 1.3	24.6 ± 0.2	1.23 ± 0.04	6.75 ± 0.05	27.28 ± 0.98	0.24 ± 0.01	3.90 ± 0.30	0.17 ± 0.02	1.30 ± 0.62
TTAG (18.8%)	10.42 ± 0.29	32.2 ± 0.4	24.7 ± 0.2	1.30 ± 0.02	6.41 ± 0.15	26.60 ± 0.55	0.24 ± 0.01	3.47 ± 0.16	0.17 ± 0.01	1.35 ± 0.10
TTGG (4.2%)	11.40 ± 0.10	33.0 ± 0.2	25.5 ± 0.0	1.20 ± 0.01	6.50 ± 0.30	28.27 ± 0.16	0.23 ± 0.01	4.05 ± 0.15	0.18 ± 0.01	1.80 ± 0.90
ANOVA/Kruskal–Wallis *p*-value	0.537	0.247	0.710	0.449	0.838	0.605	0.879	0.093	0.655	0.276

Note: D, diplotype; EW, egg weight; ELD, egg longitudinal diameter; EHD, egg horizontal diameter; ESI, egg shape index; EYH, egg yolk height; EYW, egg yolk width; EYI, egg yolk index; EYD, egg yolk diameter; EST, egg shell thickness; AH, albumen height.

**Table 5:** Association analysis of *Bsr*D I site of *VIPR-1* gene with laying performance of quail.

S	LP	*Bsr*D I (Mean ± SE)			
		GG	GT	TT	Kruskal–Wallis *p*-Value
BW (49)	F/E	4.20 ± 0.11	4.08 ± 0.04	4.01 ± 0.08	0.431
	EP (n)	38.4 ± 0.8	38.4 ± 0.5	38.2 ± 0.8	0.985
	TEM (g)	421.21 ± 13.80	422.63 ± 7.88	421.57 ± 11.57	0.985
	AES (g)	10.93 ± 0.10	10.97 ± 0.05	11.00 ± 0.08	0.991
	LR (%)	54.9 ± 1.2	54.9 ± 0.7	54.6 ± 1.1	0.959
KO (48)	F/E	3.36 ± 0.08	3.56 ± 0.05	3.41 ± 0.06	0.127
	EP (n)	50.9 ± 1.3	47.4 ± 0.7	49.7 ± 1.0	0.060
	TEM (g)	568.08 ± 14.49	535.21 ± 7.11	556.29 ± 9.92	0.060
	AES (g)	11.15 ± 0.02	11.28 ± 0.03	11.187 ± 0.03	0.231
	LR (%)	72.78 ± 1.97	67.84 ± 1.07	71.08 ± 1.44	0.074

Abbreviations: S, strain; BW, Beijing white quail; KO, Korean quail; LP, laying performance; F/E, feed to egg ratio; EP, egg production; TEM, total egg mass; AES, average egg size; LR, laying rate.

**Table 6:** Association analysis of *Hpy*CH4 IV site of *VIPR-1* gene with laying performance of quail.

S	LP	*Hpy*CH4 IV (Mean ± SE)			
		AA	AG	GG	Kruskal–Wallis *p*-Value
KO(48)	F/E	3.31 ± 0.06	3.49 ± 0.05	3.53 ± 0.06	0.510
	EP (n)	52.0 ± 1.3	48.5 ± 0.7	47.8 ± 1.0	0.089
	TEM (g)	580.05 ± 13.10	543.19 ± 7.47	540.07 ± 9.27	0.089
	AES (g)	11.15 ± 0.02	11.19 ± 0.02	11.31 ± 0.04	0.200
	LR (%)	74.28 ± 1.85	69.33 ± 1.06	68.30 ± 1.45	0.072

Notes: S, Strain; KO, Korean quail; LP, laying performance; F/E, feed to egg ratio; EP, egg production; TEM, total egg mass; AES, average egg size; LR, laying rate.

**Table 7:** Association analysis of *VIPR-1* gene haplotype combinations with laying performance of quail.

D	Traits (Mean ± SE)				
	F/E	EP (n)	TEM (g)	AES (g)	LR (%)
GGAG (4.2%)	3.49 ± 0.23	49.3 ± 3.9	550.91 ± 42.24	11.16 ± 0.04	70.50 ± 5.64
GGGG (8.3%)	3.30 ± 0.06	51.7 ± 1.2	576.67 ± 12.39	11.14 ± 0.03	73.92 ± 1.77
GTAA (4.2%)	3.25 ± 0.00	53.3 ± 0.0	593.16 ± 0.00	11.16 ± 0.00	76.14 ± 0.00
GTAG (29.2%)	3.56 ± 0.07	47.4 ± 0.9	532.43 ± 9.61	11.23 ± 0.04	67.77 ± 1.40
GTGG (27.1%)	3.60 ± 0.07	46.6 ± 1.1	529.29 ± 10.45	11.36 ± 0.06	66.64 ± 1.65
TTAA (4.2%)	3.37 ± 0.12	50.7 ± 2.6	566.95 ± 26.21	11.18 ± 0.06	72.42 ± 3.71
TTAG (18.8%)	3.39 ± 0.07	50.0 ± 1.1	558.22 ± 11.39	11.15 ± 0.02	71.50 ± 1.57
TTGG (4.2%)	3.52 ± 0.34	47.5 ± 4.8	536.94 ± 42.70	11.32 ± 0.24	67.85 ± 6.85
Kruskal–Wallis *p*-value	0.414	0.067	0.067	0.316	0.083

Note: D, diplotype; F/E, feed to egg ratio; EP, egg production; TEM, total egg mass; AES, average egg size; LR, laying rate.

## Data Availability

The data presented in this study are available from the corresponding authors upon reasonable request. The data are not publicly available due to privacy or ethical restrictions.

## Supplementary Materials

The following supporting information can be downloaded at: https://www.mdpi.com/article/10.3390/ani15101373/s1, Figure S1: Genotype frequency and allele frequency of *Bsr*D I and *Hpy*CH4 IV locus of *VIPR-1* gene; Table S1: Association analysis of *BsrD* I site of *VIPR-1* gene with egg quality of BW quail; Table S2: Association analysis of *BsrD* I site of *VIPR-1* gene with egg quality of KO quail; Table S3: Association analysis of *Hpy*CH4 IV site of *VIPR-1* gene with egg quality of KO quail; Table S4: Association analysis of haplotype combinations site of *VIPR-1* gene with egg quality of KO quail; Table S5: Association analysis of *BsrD* I site of *VIPR-1* gene with laying performance of BW quail; Table S6: Association analysis of *BsrD* I site of *VIPR-1* gene with laying performance of KO quail; Table S7: Association analysis of *Hpy*CH4 IV site of *VIPR-1* gene with laying performance of KO quail; Table S8: Association analysis of haplotype combinations site of *VIPR-1* gene with laying performance of KO quail.
