# Supplementary material for: Association of Novel Mutations in the Vasoactive Intestinal Peptide Receptor-1 Gene with Egg Shell Thickness in Three Strains of Laying-Type Quail
Source: Animals (Basel). 2025 May 9;15(10):1373. doi: 10.3390/ani15101373 (PMC12108480; doi:10.3390/ani15101373)
Supplement: Supplementary file 1 [file animals-15-01373-s001.zip › animals-3533512-supplementary.pdf]

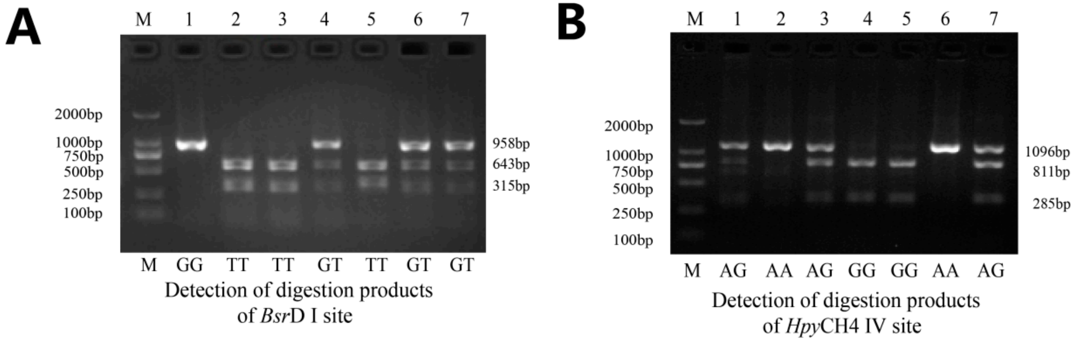

Figure S1. Genotype frequency and allele frequency of *Bsr*D I and *Hpy*CH4 IV locus of *VIPR-1* gene.

Table S1. Association analysis of *BsrD* I site of *VIPR-1* gene with egg quality of BW quail.

Trait	*Bsr*D I site g.1603402T>G	Normal distribution test Shapiro-Wilk test statistic	df	P Value		Levene test	ANOVA/Kruskal-Wallis
EW(g)	GG	0.917	8	0.404	Normal distribution		
	GT	0.969	26	0.602	Normal distribution	0.323	0.877, ANOVA
	TT	0.953	15	0.581	Normal distribution		
ELD(mm)	GG	0.93	8	0.518	Normal distribution		
	GT	0.952	26	0.253	Normal distribution	0.326	0.96, ANOVA
	TT	0.92	15	0.192	Normal distribution		
EHD(mm)	GG	0.958	8	0.79	Normal distribution		
	GT	0.936	26	0.109	Normal distribution	0.017	0.742, Kruskal-Wallis
	TT	0.945	15	0.456	Normal distribution		
ESI	GG	0.873	8	0.16	Normal distribution		
	GT	0.973	26	0.707	Normal distribution	0.358	0.833, ANOVA
	TT	0.974	15	0.916	Normal distribution		
EYH(mm)	GG	0.869	8	0.146	Normal distribution		
	GT	0.97	26	0.624	Normal distribution	0.213	0.5, ANOVA
	TT	0.961	15	0.706	Normal distribution		
EYD(mm)	GG	0.947	8	0.684	Normal distribution		
	GT	0.921	26	0.048	Non-normal distribution	x	0.575, Kruskal-Wallis
	TT	0.93	15	0.272	Normal distribution		
EYI	GG	0.597	8	<0.001	Non-normal distribution		
	GT	0.982	26	0.909	Normal distribution	x	0.223, Kruskal-Wallis
	TT	0.912	15	0.147	Normal distribution		
EYW(g)	GG	0.902	8	0.301	Normal distribution	0.091	0.888, ANOVA

	GT	0.968	26	0.575	Normal distribution				
	TT	0.972	15	0.886	Normal distribution				
EST(cm)	GG	0.808	8	0.035	Non-normal distribution				
	GT	0.967	26	0.547	Normal distribution	x	0.043, Kruskal-Wallis	GG-GT, 0.896<br>GT-TT, 0.039<br>TT-GG, 1.000	Dun-Bonferroni
	TT	0.93	15	0.272	Normal distribution				
AH(mm)	GG	0.918	8	0.41	Normal distribution				
	GT	0.97	26	0.616	Normal distribution	0.362	0.922, ANOVA		
	TT	0.962	15	0.734	Normal distribution				

Traits including EYD, EYI, and EST in the BW strain at the g.1603402T>G locus deviated from normality (P < 0.05, Table S1). The nonparametric test did not show significant differences among the genotypes for EYD and EYI (P > 0.05). Only EST significantly differed among the genotypes (P = 0.043). Multiple comparison tests were performed using Dun-Bonferroni corrected P values. The TG genotype of the g.1603402T>G site showed significantly higher EST in the BW population (P < 0.05). The remaining seven traits conformed to a normal distribution and were subjected to a homogeneity of variance test; the variances of EHD were inconsistent (P < 0.05). The nonparametric test did not show significant differences among the genotypes for EHD (P > 0.05). The remaining six traits were analyzed by ANOVA, and there were not significant differences (P > 0.05) among the genotypes in the BW population. X: Populations that were not normally distributed did not undergo a chi-square test, and non-parametric tests were used directly to test whether there was a difference among the genotypes.

Table S2. Association analysis of *BsrD* I site of *VIPR-1* gene with egg quality of KO quail.

Trait	*Bsr*D I site g.1603402T>G	Normal distribution test Shapiro-Wilk test statistic	df	P Value		Levene test	ANOVA/Kruskal-Wallis
EW(g)	GG	0.86	6	0.191	Normal distribution		
	GT	0.981	29	0.856	Normal distribution	0.505	0.795, ANOVA
	TT	0.957	13	0.704	Normal distribution		
ELD(mm)	GG	0.824	6	0.096	Normal distribution		
	GT	0.967	29	0.493	Normal distribution	0.82	0.697, ANOVA
	TT	0.972	13	0.913	Normal distribution		
EHD(mm)	GG	0.868	6	0.216	Normal distribution		
	GT	0.976	29	0.74	Normal distribution	0.262	0.896, ANOVA
	TT	0.92	13	0.25	Normal distribution		
ESI	GG	0.852	6	0.162	Normal distribution		
	GT	0.937	29	0.086	Normal distribution	0.953	0.773, ANOVA
	TT	0.902	13	0.144	Normal distribution		
EYH(mm)	GG	0.945	6	0.698	Normal distribution		
	GT	0.981	29	0.871	Normal distribution	0.005	0.776, Kruskal-Wallis
	TT	0.939	13	0.442	Normal distribution		
EYD(mm)	GG	0.917	6	0.484	Normal distribution		
	GT	0.981	29	0.854	Normal distribution	**0.65**	0.533, ANOVA
	TT	0.905	13	0.154	Normal distribution		
EYI	GG	0.862	6	0.195	Normal distribution		
	GT	0.959	29	0.308	Normal distribution	0.009	0.316, Kruskal-Wallis
	TT	0.956	13	0.696	Normal distribution		
EYW(g)	GG	0.974	6	0.917	Normal distribution	0.091	0.568, ANOVA

	GT	0.955	29	0.24	Normal distribution		
	TT	0.893	13	0.106	Normal distribution		
EST(cm)	GG	0.945	6	0.701	Normal distribution		
	GT	0.928	29	0.048	Normal distribution	0.427	0.825, ANOVA
	TT	0.958	13	0.718	Normal distribution		
AH(mm)	GG	0.933	6	0.606	Normal distribution		
	GT	0.9	29	0.01	Non-normal distribution	x	0.939, Kruskal-Wallis
	TT	0.938	13	0.426	Normal distribution		

The AH in the KO strain at the g.1603402T>G locus deviated from normality ($P < 0.05$, Table S2). The nonparametric test did not show significant differences among the genotypes for AH ($P > 0.05$). The remaining nine traits conformed to a normal distribution for the homogeneity of variance test. The homogeneity of variance test of EYH and EYI was inconsistent ($P < 0.05$). The nonparametric test did not show significant differences among the genotypes for EYH and EYI ($P > 0.05$). ANOVA analyzed the remaining seven traits, and there were no significant differences ($P > 0.05$) among the genotypes in the KO population. x: Populations that were not normally distributed did not undergo a chi-square test, and non-parametric tests were used to directly test whether there was a difference among the genotypes.

Table S3. Association analysis of *Hpy*CH4 IV site of *VIPR-1* gene with egg quality of KO quail.

Trait	*Hpy*CH4 IV site	Normal distribution test				Levene test	ANOVA/Kruskal-Wallis
		Shapiro-Wilk test					
	g.1614884A > G	statistic	df	P Value			
EW(g)	AA	0.954	4	0.74	Normal distribution		
	AG	0.968	25	0.605	Normal distribution	0.584	0.665, ANOVA
	GG	0.962	19	0.607	Normal distribution		
ELD(mm)	AA	0.885	4	0.362	Normal distribution		
	AG	0.971	25	0.672	Normal distribution	0.286	0.154, ANOVA
	GG	0.955	19	0.485	Normal distribution		
EHD(mm)	AA	0.883	4	0.351	Normal distribution		
	AG	0.979	25	0.875	Normal distribution	0.227	0.975, ANOVA
	GG	0.959	19	0.55	Normal distribution		
ESI	AA	0.81	4	0.122	Normal distribution		
	AG	0.962	25	0.459	Normal distribution	0.286	0.056, ANOVA
	GG	0.92	19	0.114	Normal distribution		
EYH(mm)	AA	0.838	4	0.189	Normal distribution		
	AG	0.957	25	0.359	Normal distribution	0.339	0.367, ANOVA
	GG	0.952	19	0.431	Normal distribution		
EYD(mm)	AA	0.883	4	0.353	Normal distribution		
	AG	0.972	25	0.687	Normal distribution	0.149	0.956, ANOVA
	GG	0.952	19	0.423	Normal distribution		
EYI	AA	0.989	4	0.951	Normal distribution		
	AG	0.978	25	0.844	Normal distribution	0.476	0.753, ANOVA
	GG	0.954	19	0.458	Normal distribution		
EYW(g)	AA	0.971	4	0.85	Normal distribution	x	0.151, Kruskal-Wallis

	AG	0.896	25	0.015	Non-normal distribution		
	GG	0.934	19	0.205	Normal distribution		
EST(cm)	AA	0.895	4	0.408	Normal distribution		
	AG	0.945	25	0.191	Normal distribution	x	0.155, Kruskal-Wallis
	GG	0.891	19	0.034	Non-normal distribution		
AH(mm)	AA	0.827	4	0.161	Normal distribution		
	AG	0.894	25	0.014	Non-normal distribution	x	0.568, Kruskal-Wallis
	GG	0.916	19	0.096	Normal distribution		

Traits including EYW, EST, and AH in the KO strain at the g.1614884A > G locus deviated from normality ($P < 0.05$, Table S3).The nonparametric test did not show significant differences among the genotypes for EYW, EST, and AH ($P > 0.05$). The remaining seven traits conformed to a normal distribution and were subjected to the homogeneity of variance test; the variances of seven traits were consistent ($P > 0.05$). These traits were analyzed by ANOVA, and the results were not significant differences ($P > 0.05$, Table S3) among all genotypes in KO population. x: Populations that were not normally distributed did not undergo a chi-square test, and non-parametric tests were used to directly test whether there was a difference among the genotypes.

Table S4. Association analysis of haplotype combinations site of *VIPR-1* gene with egg quality of KO quail.

Trait	haplotype	Normal distribution test Shapiro-Wilk test statistic	df	P Value		Levene test	ANOVA/Kruskal-Wallis
EW(g)	GGAG						
	GGGG	0.716	4	0.017	Non-normal distribution		
	GTAA						
	GTAG	0.897	14	0.101	Normal distribution	x	0.537, Kruskal-Wallis
	GTGG	0.971	13	0.908	Normal distribution		
	TTAA						
	TTAG	0.978	9	0.951	Normal distribution		
	TTGG						
ELD(mm)	GGAG						
	GGGG	0.961	4	0.783	Normal distribution		
	GTAA						
	GTAG	0.917	14	0.199	Normal distribution	0.016	0.247, Kruskal-Wallis
	GTGG	0.965	13	0.829	Normal distribution		
	TTAA						
	TTAG	0.972	9	0.915	Normal distribution		
	TTGG						
EHD(mm)	GGAG						
	GGGG	0.796	4	0.096	Normal distribution		
	GTAA						
	GTAG	0.895	14	0.096	Normal distribution	0.284	0.710, ANOVA
	GTGG	0.974	13	0.935	Normal distribution		
	TTAA						

	TTAG	0.922	9	0.411	Normal distribution		
	TTGG						
ESI	GGAG						
	GGGG	0.974	4	0.864	Normal distribution		
	GTAA						
	GTAG	0.959	14	0.699	Normal distribution	x	0.449, Kruskal-Wallis
	GTGG	0.864	13	0.043	Non-normal distribution		
	TTAA						
	TTAG	0.85	9	0.075	Normal distribution		
	TTGG						
EYH(mm)	GGAG						
	GGGG	0.827	4	0.161	Normal distribution		
	GTAA						
	GTAG	0.967	14	0.837	Normal distribution	0.019	0.838, Kruskal-Wallis
	GTGG	0.935	13	0.394	Normal distribution		
	TTAA						
	TTAG	0.935	9	0.526	Normal distribution		
	TTGG						
EYD(mm)	GGAG						
	GGGG	0.948	4	0.704	Normal distribution		
	GTAA						
	GTAG	0.962	14	0.759	Normal distribution	0.057	0.605, ANOVA
	GTGG	0.94	13	0.457	Normal distribution		
	TTAA						
	TTAG	0.94	9	0.581	Normal distribution		
	TTGG						

EYI	GGAG						
	GGGG	0.797	4	0.097	Normal distribution		
	GTAA						
	GTAG	0.979	14	0.969	Normal distribution	0.137	0.879, ANOVA
	GTGG	0.902	13	0.144	Normal distribution		
	TTAA						
	TTAG	0.947	9	0.656	Normal distribution		
	TTGG						
EYW(g)	GGAG						
	GGGG	0.863	4	0.272	Normal distribution		
	GTAA						
	GTAG	0.886	14	0.071	Normal distribution	0.552	0.093, ANOVA
	GTGG	0.935	13	0.397	Normal distribution		
	TTAA						
	TTAG	0.892	9	0.208	Normal distribution		
	TTGG						
EST(cm)	GGAG						
	GGGG	0.884	4	0.356	Normal distribution		
	GTAA						
	GTAG	0.894	14	0.091	Normal distribution	x	0.655, Kruskal-Wallis
	GTGG	0.866	13	0.047	Non-normal distribution		
	TTAA						
	TTAG	0.975	9	0.936	Normal distribution		
	TTGG						
AH(mm)	GGAG					x	0.276, Kruskal-Wallis
	GGGG	0.916	4	0.516	Normal distribution		

GTAA				
GTAG	0.833	14	0.013	Normal distribution
GTGG	0.912	13	0.196	Normal distribution
TTAA				
TTAG	0.919	9	0.387	Normal distribution
TTGG				

Traits including EW, ESI, EST, and AH in the KO strain at the haplotype combinations deviated from normality ($P < 0.05$, Table S4). The nonparametric test did not show significant differences among the haplotype combinations for EW, ESI, EST and AH ($P > 0.05$). The remaining six traits conformed to a normal distribution and were subjected to a homogeneity of variance test, the variances of ELD and EYH were inconsistent ($P < 0.05$). The nonparametric test did not show significant differences among the haplotype combinations for ELD and EYH ($P > 0.05$). The remaining four traits were analyzed by ANOVA, and the results were not significant differences ($P > 0.05$) among all haplotype combinations in the KO population. x: Populations that were not normally distributed did not undergo a chi-square test, and non-parametric tests were used to directly test whether there was a difference among the genotypes.

Table S5. Association analysis of *BsrD* I site of *VIPR-1* gene with laying performance of BW quail.

Trait	*Bsr*D I site g.1603402T>G	Normal distribution test Shapiro-Wilk test statistic	df	P Value		Levene test	ANOVA/Kruskal-Wallis
F/E	GG	0.79	8	0.022	Non-normal distribution		
	GT	0.865	26	0.003	Non-normal distribution	x	0.431, Kruskal-Wallis
	TT	0.834	15	0.01	Non-normal distribution		
AES(g)	GG	0.815	8	0.041	Non-normal distribution		
	GT	0.843	26	0.001	Non-normal distribution	x	0.991, Kruskal-Wallis
	TT	0.798	15	0.003	Non-normal distribution		
EP(n)	GG	0.747	8	0.008	Non-normal distribution		
	GT	0.822	26	0	Non-normal distribution	x	0.985, Kruskal-Wallis
	TT	0.796	15	0.003	Non-normal distribution		
TEM(g)	GG	0.78	8	0.017	Non-normal distribution		
	GT	0.842	26	0.001	Non-normal distribution	x	0.985, Kruskal-Wallis
	TT	0.799	15	0.004	Non-normal distribution		
LR(%)	GG	0.747	8	0.008	Non-normal distribution		
	GT	0.822	26	0	Non-normal distribution	x	0.959, Kruskal-Wallis
	TT	0.796	15	0.003	Non-normal distribution		

All traits in the BW strain at the g.1603402T>G locus deviated from normality ($P < 0.05$, Table S5). The nonparametric test did not show significant differences among the genotypes for all traits ($P > 0.05$) in the BW population. x: Populations that were not normally distributed did not undergo a chi-square test, and non-parametric tests were used to directly test whether there was a difference among the genotypes.

Table S6. Association analysis of *BsrD* I site of *VIPR-1* gene with laying performance of KO quail.

Trait	*Bsr*D I site g.1603402T>G	Normal distribution test Shapiro-Wilk test statistic	df	P Value		Levene test	ANOVA/Kruskal-Wallis
F/E	GG	0.802	6	0.062	Normal distribution		
	GT	0.84	29	<0.001	Non-normal distribution	x	0.127, Kruskal-Wallis
	TT	0.838	13	0.02	Non-normal distribution		
AES(g)	GG	0.885	6	0.294	Normal distribution		
	GT	0.77	29	<0.001	Non-normal distribution	x	0.231, Kruskal-Wallis
	TT	0.728	13	0.001	Non-normal distribution		
EP(n)	GG	0.766	6	0.029	Non-normal distribution		
	GT	0.852	29	0.001	Non-normal distribution	x	0.060, Kruskal-Wallis
	TT	0.846	13	0.025	Non-normal distribution		
TEM(g)	GG	0.783	6	0.041	Non-normal distribution		
	GT	0.833	29	<0.001	Non-normal distribution	x	0.060, Kruskal-Wallis
	TT	0.853	13	0.031	Non-normal distribution		
LR(%)	GG	0.766	6	0.029	Non-normal distribution		
	GT	0.852	29	<0.001	Non-normal distribution	x	0.074, Kruskal-Wallis
	TT	0.846	13	0.025	Non-normal distribution		

All traits in the KO strain at the g.1603402T>G locus deviated from normality ($P < 0.05$, Table S6). The nonparametric test did not show significant differences among the genotypes for all traits ($P > 0.05$) in the KO population. x: Populations that were not normally distributed did not undergo a chi-square test, and non-parametric tests were used to directly test whether there was a difference among the genotypes.

Table S7. Association analysis of *Hpy*CH4 IV site of *VIPR-1* gene with laying performance of KO quail.

Trait	*Hpy*CH4 IV *site* g.1614884A > G	Normal distribution test Shapiro-Wilk test statistic	df	P Value		Levene test	ANOVA/Kruskal-Wallis
F/E	GG	0.802	6	0.062	Normal distribution		
	GT	0.84	29	<0.001	Non-normal distribution	x	0.127, Kruskal-Wallis
	TT	0.838	13	0.02	Non-normal distribution		
AES(g)	GG	0.885	6	0.294	Normal distribution		
	GT	0.77	29	<0.001	Non-normal distribution	x	0.231, Kruskal-Wallis
	TT	0.728	13	0.001	Non-normal distribution		
EP(n)	GG	0.766	6	0.029	Non-normal distribution		
	GT	0.852	29	0.001	Non-normal distribution	x	0.060, Kruskal-Wallis
	TT	0.846	13	0.025	Non-normal distribution		
TEM(g)	GG	0.783	6	0.041	Non-normal distribution		
	GT	0.833	29	<0.001	Non-normal distribution	x	0.060, Kruskal-Wallis
	TT	0.853	13	0.031	Non-normal distribution		
LR(%)	GG	0.766	6	0.029	Non-normal distribution		
	GT	0.852	29	<0.001	Non-normal distribution	x	0.074, Kruskal-Wallis
	TT	0.846	13	0.025	Non-normal distribution		

All traits in the KO strain at the g.1614884A > G locus deviated from normality ($P < 0.05$, Table S7). The nonparametric test did not show significant differences among the genotypes for all traits ($P > 0.05$) in the KO population. x: Populations that were not normally distributed did not undergo a chi-square test, and non-parametric tests were used to directly test whether there was a difference among the genotypes.

Table S8. Association analysis of haplotype combinations site of *VIPR-1* gene with laying performance of KO quail.

Trait	haplotype	Normal distribution test Shapiro-Wilk test				Levene test	ANOVA/Kruskal-Wallis
		statistic	df	P Value			
F/E	GGAG						
	GGGG	0.829	4	0.164	Normal distribution		
	GTAA						
	GTAG	0.815	14	0.008	Non-normal distribution	x	0.414, Kruskal-Wallis
	GTGG	0.8	13	0.007	Non-normal distribution		
	TTAA						
	TTAG	0.817	9	0.032	Non-normal distribution		
	TTGG						
AES(g)	GGAG						
	GGGG	0.866	4	0.284	Normal distribution		
	GTAA						
	GTAG	0.729	14	0.001	Non-normal distribution	x	0.316, Kruskal-Wallis
	GTGG	0.774	13	0.003	Non-normal distribution		
	TTAA						
	TTAG	0.843	9	0.062	Normal distribution		
	TTGG						
EP(n)	GGAG						
	GGGG	0.759	4	0.047	Non-normal distribution		
	GTAA					x	0.067, Kruskal-Wallis
	GTAG	0.849	14	0.022	Non-normal distribution		
	GTGG	0.804	13	0.008	Non-normal distribution		
	TTAA						

	TTAG	0.818	9	0.033	Non-normal distribution		
	TTGG						
TEM(g)	GGAG						
	GGGG	0.795	4	0.093	Normal distribution		
	GTAA						
	GTAG	0.812	14	0.007	Non-normal distribution	x	0.067, Kruskal-Wallis
	GTGG	0.811	13	0.009	Non-normal distribution		
	TTAA						
	TTAG	0.839	9	0.057	Normal distribution		
	TTGG						
LR(%)	GGAG						
	GGGG	0.759	4	0.047	Non-normal distribution		
	GTAA						
	GTAG	0.849	14	0.022	Non-normal distribution	x	0.083, Kruskal-Wallis
	GTGG	0.804	13	0.008	Non-normal distribution		
	TTAA						
	TTAG	0.818	9	0.033	Non-normal distribution		
	TTGG						

All traits in the KO strain at the haplotype combinations deviated from normality ($P < 0.05$, Table S8). The nonparametric test did not show significant differences among the haplotype combinations for all traits ($P > 0.05$) in the KO population. x: Populations that were not normally distributed did not undergo a chi-square test, and non-parametric tests were used to directly test whether there was a difference among the haplotype combinations.
